# The involvement of DARPP-32 in the pathophysiology of schizophrenia

**DOI:** 10.18632/oncotarget.17339

**Published:** 2017-04-21

**Authors:** Haitao Wang, Mohd Farhan, Jiangping Xu, Philip Lazarovici, Wenhua Zheng

**Affiliations:** ^1^ Faculty of Health Sciences, University of Macau, Taipa, Macau, China; ^2^ School of Pharmaceutical Sciences, Southern Medical University, Guangzhou, China; ^3^ School of Pharmacy Institute for Drug Research, Faculty of Medicine, The Hebrew University of Jerusalem, Jerusalem, Israel

**Keywords:** schizophrenia, DARPP-32, dopamine, cAMP, glutamate

## Abstract

Schizophrenia is one of the most devastating heterogeneous psychiatric disorders. The dopamine hypothesis is the longest standing pathoetiologic theory of schizophrenia based on neurochemical evidences of elevated brain striatal dopamine synthesis capacity and increased dopamine release in response to stress. Dopamine and cyclic AMP-regulated phosphoprotein of relative molecular mass 32,000 (DARPP-32) is a cytosolic protein highly enriched in the medium spiny neurons of the neostriatum, considered as the most important integrator between the cortical input and the basal ganglia, and associated with motor control. Accumulating evidences has indicated the involvement of DARPP-32 in the development of schizophrenia; i. DARPP-32 phosphorylation is regulated by several neurotransmitters, including dopamine and glutamate, neurotransmitters implicated in schizophrenia pathogenesis; ii. decrease of both total and phosphorylated DARPP-32 in the prefrontal cortex are observed in schizophrenic animal models; iii. postmortem brain studies indicated decreased expression of DARPP-32 protein in the superior temporal gyrus and dorsolateral prefrontal cortex in patients with schizophrenia; iv. DARPP-32 phosphorylation is increased upon therapy with antipsychotic drugs, such as haloperidol and risperidone which improve behavioral performance in experimental animal models and patients; v. Genetic analysis of the gene coding for DARPP-32 propose an association with schizophrenia. Cumulatively, these findings implicate DARPP-32 protein in schizophrenia and propose it as a potential therapeutic target. Here, we summarize the possible roles of DARPP-32 during the development of schizophrenia and make some recommendations for future research. We propose that DARPP-32 and its interacting proteins may serve as potential therapeutic targets in the treatment of schizophrenia.

## INTRODUCTION

Schizophrenia is a severe mental disorder with a worldwide lifetime risk of approximately 1% of the population. This schizophrenic syndrome is characterized by cumulative psychiatric deficits including positive symptoms (e.g. delusions, hallucinations, and disorganized thought speech and /or behavior) and negative symptoms (e.g. affective flattening, lack of motivation, poor relatedness and social withdrawal) causing impairments of working and semantic memory [1, 2]. The accurate causes and mechanisms of this idiopathic disorder are still unknown. It is hypothesized that genetic, environmental insults and vulnerability factors combine and interact with each other at critical periods of neurodevelopment and thus, alter the morphologies and physiological functions of neurons in specific brain regions [3–5], contributing to this mental disorder. Schizophrenic patients also suffer disproportionately from mood symptoms and are characterized by a significant risk of suicide [6]. Schizophrenia has been also considered as a neurodevelopmental disorder [7, 8].

To date, many hypotheses have been proposed to explain the disease, including dopamine hypothesis [9], inflammation and the neural diathesis-stress hypothesis [10], neurodevelopmental hypothesis [11] and glutamate hypothesis [12]. Among these multiple hypotheses, the dopamine hypothesis is the most accepted and longest standing pathoetiologic theory of schizophrenia [13], for more than several decades. The dopamine hypothesis was established based on several lines of evidences: i. clinical studies found that dopaminergic agonists could induce psychosis in normal individuals and could worsen psychosis in patients suffering from schizophrenia; ii. antipsychotic drugs blocked the dopamine receptors and their potency is linked to their affinity for dopamine D2 receptors, linking this receptor subtype to phenotype traits observed in patients; iii. postmortem studies provided direct evidence for elevated levels of dopamine, its metabolites, and its receptors in the striatum of schizophrenic patients [13].

Brain dopaminergic neuronal pathways are critical in maintaining multiple functions, including movement, rewards and stress [14]. Changes in neuronal circuits function can be regulated by dopaminergic receptor-receptor homologous and heterologous interactions, such as interaction between dopamine 1-like receptor (D1R) and dopamine 2-like receptor (D2R), or interaction with other signaling proteins inside the cell, or cross talk with heterologous receptors [15]. Dopamine receptors are divided into two families, D1R and D2R [16]. Both families belong to G-protein coupled receptors (GPCRs), which are linked to adenylyl cyclase (AC), that converse adenosine triphosphate (ATP) to cyclic adenosine monophosphate (cAMP). Specifically, D1Rs are coupled to Gs (stimulatory G-protein) and D2Rs are coupled to Gi (inhibitory G-protein). Activation of Gs-protein increases cAMP, while activation of Gi leads to the reduction of cAMP level [17]. cAMP in turn, activates cAMP-dependent protein kinase A which phosphorylates a large number of protein substrates, such as phospholipases, protein kinases, tyrosine kinase receptors and ion channels located within the plasma membrane [18].

One well-studied target for the phosphorylation activity of dopamine receptors is “Dopamine and cAMP-regulated phosphoprotein” of relative molecular mass 32,000 daltons (DARPP-32). DARPP-32, also known as phosphoprotein phosphatase-1 regulatory subunit 1B (PPP1R1B), was initially found as a substrate of protein kinase A (PKA), activated by dopamine receptors D1R in the brain neostriatum [19]. In the central nervous system (CNS), DARPP-32 is expressed in medium spiny neurons (MSNs) that also co-express dopamine D1R [20] and containseveral sites forregulatory phosphorylations. Dopamine stimulates phosphorylation of DARPP-32 substrate at Thr34 residue by binding to D1R and subsequent activation of PKA [21]. DARPP-32, when phosphorylated at another site, Thr75, by cyclin-dependent kinase 5 (CDK5), is converted into an inhibitor of PKA [22]. Targeted deletion of the DARPP-32 gene in the mouse brain produced an altered biochemical and behavioral phenotype similar to schizophrenia [23]. Dopamine exerts a bidirectional control on the state of phosphorylation of DARPP-32. On one hand, activation of D1R increased the phosphorylation of DARPP-32 and activation of D2R decreased basal phosphorylation of DARPP-32 [24]. On the other hand, DARPP-32 is involved in regulating dopamine receptor-induced transcription of several physiologically important genes in striatum and in globus pallidus, such as such as *c-fos* and *NGFI-A* [25]. The link between DARPP-32 and mental disorders, especially schizophrenia, has aroused extensive interest. Here, we review some of the current evidences and new ideas that are proposing DARPP-32 involvement in schizophrenia and suggest a few possibilities for future investigations.

### Expression of darpp-32 in the central nervous system

Neuronal enzymes and their regulators and substrates play a fundamental role in brain function. On one hand, they are under the control of gene transcription, but they are also regulated by post-translational activities, such as phosphorylation and translocation to sub-cellular organelles. DARPP-32 is expressed at low levels prenatally, with the onset at day 14 of gestation in the rostral part of the primordial olfactory tubercle [26]. On day 18 of gestation, DARPP-32-immunoreactive neurons are first visible in the brain nucleus accumbens which, together with the olfactory tubercle, forms the ventral striatum, is part of the basal ganglia involved in cognitive processing of aversion, motivation, reward and addiction. The expression of DARPP-32 is gradually increased during brain development with strongly labeled neurons observed at the day of birth, reaching maximal expression 3 weeks postnatally [26]. Within the brain, DARPP-32 is mainly expressed in caudate, putamen, nucleus accumbens, cerebral and cerebellar cortex, all of these regions receiving dopaminergic projections and therefore involved in the pathogenesis of the schizophrenic disease [27]. At a cellular level, DARPP-32 protein is expressed in neuronal cell bodies and dendrites [28]. Specifically, DARPP-32 is present in the subclasses of dopaminergic neurons containing D1R, so, DARPP-32 is deemed to be a representative marker for striatal projection neurons [29].

**Figure 1:**
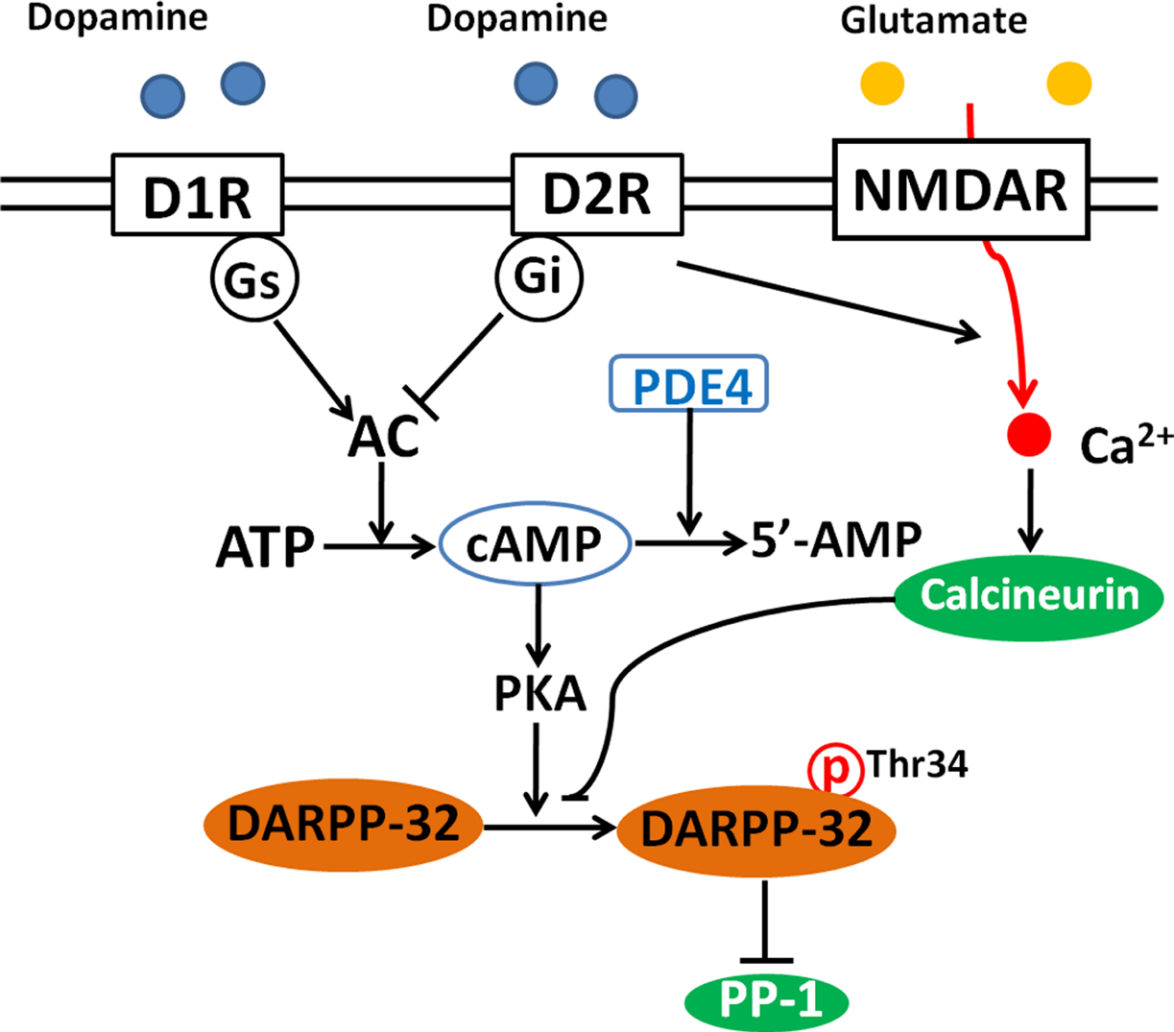
Modulation of DARPP-32 by dopamine and NMDA receptors Binding of dopamine with D1 receptors (D1R) activates adenylate cyclase (AC) and increases intracellular cAMP, leading to the activation of PKA and the subsequent phosphorylation of DARPP-32 at Thr34. Activation of D2 receptors (D2R) inhibits AC, decreases the level of cAMP and DARPP-32 phosphorylation. Activation of NMDA receptor leads to increased intracellular calcium and activates calcium-dependent calcineurin, which promotes the dephosphorylation of DARPP-32 at Thr34. Phosphodiesterase 4 (PDE4) is an enzyme responsible for the hydrolysis of cAMP. Inhibition of PDE4 is supposed to enhances cAMP and thereby activates PKA.

Glutamatergic neurotransmission, including NMDA and other glutamatergic receptors have been involved in schizophrenia [30]. There are evidences that NMDA hypofunction disrupts the inhibitory/excitatory homeostasis and thereby leads to enhanced dopamine release from presynaptic dopaminergic neurons [13, 31]. Therefore is not surprising that DARPP-32 also acts as an integrator of dopaminergic and glutamatergic input signaling [20]. Dopamine receptors D1R and the N-methyl-D-aspartate glutamate receptors (NMDAR) are involved in the prefrontal dysfunction linked with schizophrenic illness [31]. Interestingly, these two receptors antagonistically modulate the phosphorylation of DARPP-32 [32], probably attributed to the different G proteins coupling these receptors to the adenylate cyclase and regulation the concentration of cAMP and activity of PKA. Modulation of DARPP-32 protein phosphorylation by dopamine and NMDA receptors is presented in Figure 1. The physiological consequences of this antagonistic modulation of DARPP-32 is expressed on its critical role in synaptic plasticity [33], and glutamatergic transmission [33] in which activation of DARPP-32 is required for the induction of both long-term depression (LTD) and long-term potentiation (LTP) [33] basic processes involved in learning and memory [34].

In the mammalian brain, PPP1R1B gene encodes two different transcripts of DARPP-32 protein, a full length DARPP-32 and a truncated DARPP-32. Truncated transcript encodes a protein that lacks the first 36 residues of N-terminus, which contains the phosphorylation site Thr 34 and the PP-1 interacting domain [35], and therefore cannot be phosphorylated by PKA and has no effect on PP-1. Truncated DARPP-32 form is only detected in adult brain striatal neurons in which the protein expression levels is higher than those of the full length DARPP-32 [35]. As truncated DARPP-32 lacks the PP-1 interacting domain, the biological functions of full length and truncated DARPP-32 proteins might be different. Truncated DARPP isoform in MSNs might be necessary for the specification and connectivity establishment of the different neuronal subtypes, but less required for mature circuitry formation [35]. The protein level of truncated DARPP-32 is significantly increased in the dorsolateral prefrontal cortex and caudate of patients with schizophrenia [36, 37]. The mechanism and role of the late developmental expression of truncated DARPP-32 in the CNS is yet unknown, and deserves further exploration.

### Regulation of darpp-32 by kinases and phosphatases in the central nervous system

**Figure 2:**
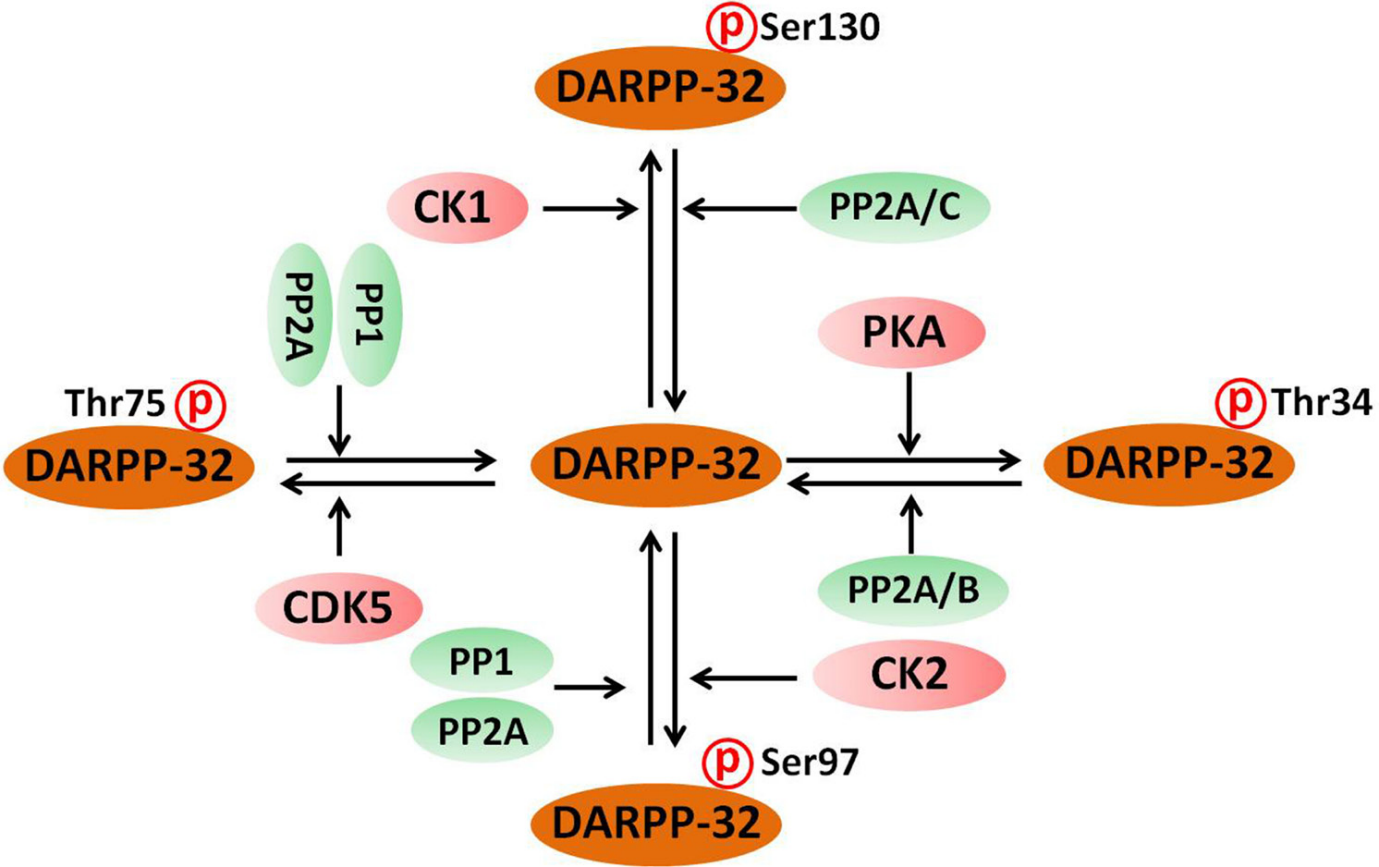
Phosphorylation of DARPP-32 by protein kinases and phosphatases DARPP-32 is phosphorylated by PKA, CDK5, CK1 and CK2 protein kinases at different phosphorylation sites. On the other hand, DARPP-32 is dephosphorylated by protein phosphatase 1 (PP-1) protein phosphatase 2A (PP2A) and calcineurin (PP2B).

The activity of DARPP-32 depends on the state of phosphorylation at multiple regulatory sites, including Thr34, Thr75, Ser97 and Ser130. Phosphorylation of DARPP-32 by protein kinases and phosphatases is schematically presented in Figure 2. DARPP-32 can be activated by various neurotransmitters, including dopamine, glutamate, serotonin and adenosine [20, 38–40]. Dopamine regulates the phosphorylation of DARPP-32 through binding to D1R receptors which activates PKA. PKA in turn promotes the phosphorylation of DARPP-32 at Thr34, leading to its conversion into an inhibitor of protein phosphatase 1(PP-1) [21]. PP-1 dephosphorylates a large number of neuronal phosphoproteins, and therefore DARPP-32-mediated inhibition of PP-1, inhibited or reduced the phosphorylation of proteins involved in synaptic function and plasticity [20]. Similar to activation of D1R, binding of adenosine to A2A receptor also leads to increased level of cAMP, activation of PKA and increased phosphorylation of DARPP-32 at Thr34 [41]. Since activation of adenosine A2A receptors can enhance the release of several neurotransmitters including dopamine [42], this common phosphorylation effect may amplify DARPP-32 synaptic activity. It is rather remarkable that activation of D1R increases AC activity and thereby increasing DARPP-32 phosphorylation at Thr34 [24], while activation of the D2R on neostriatal neurons decreases AC activity and the formation of cAMP, and therefore decreases DARPP-32 phosphorylation at Thr34 [43]. When D2R receptors are co-expressed with adenosine A2A receptor, it can also result in a strong decrease of cAMP levels, inhibiting the activity of both PKA and in phosphorylation of DARPP-32 at Thr34 [44]. The opposite regulation exerted by A2A receptors and D2R receptors on DARPP-32 phosphorylation could explain some of the antagonistic actions of dopamine and adenosine in schizoprenia.

It was also reported that activation of D2R increased the neuronal influx of Ca^2+^ and the activity of calcium-dependent protein phosphatase PP2B (named calcineurin), leading to dephosphorylation of DARPP-32 [24]. Activation of the N-methyl-D-aspartate glutamate receptors also leads to the activation of calcineurin and reverses the cAMP-induced phosphorylation of DARPP-32 in striatum [45]. Blocking the activation of D2R in the striatum, promotes the phosphorylation of DARPP-32 at Thr34 and can be reversed by inhibiting either D1R or A2A receptors [46]. Adenosine A2A receptors (A2AR) located in the postsynaptic membrane in the striato-pallidal GABAergic neurons, antagonize the biological function of dopamine D2 receptors, while A2AR receptors expressed presynaptically at cortico-striatal terminals usually promotes glutamate release. Therefore it is tempting to propose that A2A receptors cause fine-tune striatal activity by integrating DARPP-32 mediated dopaminergic and glutamatergic signaling [41].

Besides phosphorylation at Thr34 by PKA, DARPP-32 is also phosphorylated by cyclin-dependent kinase 5 (CDK5) at Thr75, converting DARPP-32 into a PKA inhibitor [22]. Activation of NMDA receptors decreases the phosphorylation of DARPP-32 at Thr75, and this effect is mediated through calcium-dependent activation of protein phosphatase-2A (PP2A) [47, 48]. Hence, glutamate increases the phoshorylation and activity of DARPP-32 at Thr34 or Thr75 through activating different calcium-dependent protein phosphatases. Another complexity of calcium regulation is the calcium activation effect on protein kinase C (PKC) and Ca^2+^/calmodulin-dependent protein kinase II (CaMK II). Activation of these kinases also results in DARPP32-Thr75 phosphorylation [49]. Therefore, different protein kinases and phosphatases activated by glutamate receptors dynamically modulate the phosphorylation of DARPP32-Thr75 site [49].

Casein kinase 1 (CK1) and casein kinase 2 (CK2) are additional protein kinases inducing the phosphorylation of DARPP-32. CK2 phosphorylates DARPP-32 at Ser97, while CK1's phosphorylation site is Ser130 [50]. Phosphorylation of DARPP-32 at Ser97 promotes the phosphorylation of DARPP-32 at Thr34 by cAMP-dependent PKA [51] and phosphorylation of Ser137 by CK1 decreases dephosphorylation of Thr34 by calcineurin [52]. Phosphorylation of DARPP-32 by CK1 and CK2 occurs in response to increased levels of cAMP [51]. It is also known that activation of D1R/PKA signaling activates PP2A/B56δ, and promotes the dephosphorylation of DARPP-32 at Ser97, eventually reducing the export of DARPP-32 out of the nucleus [53]. Cytonuclear trafficking of DARPP-32 may be important to the biological functions of dopamine on striatal neurons. Following activation of D1R, DARPP-32 is accumulated in the nucleus of dopaminergic neurons and this facilitates the phosphorylation of histone H3 at Ser10, a site dephosphorylated by PP1, suggesting that DARPP-32 is involved in the regulation of alternative splicing or gene expression [54]. Recently, DARPP-32 Ser97 phosphorylation was reported to mediate the binding of DARPP-32 to β-adducin, an actin-capping protein that stabilizes the neuronal cytoskeleton, and this effect was reinforced by DARPP-32 Thr75 phosphorylation [55]. As β-adducin increases the density of neuronal dendritic spines under the conditions of novel enriched environment, DARPP-32 is viewed as a regulator of rapid neuronal modifications induced by environment, to enforce learning and memory [55].

The phosphorylation state of DARPP-32 is determined by both protein kinases and phosphatases. As shown in Figure 2, there are four major phosphorylation sites for DARPP-32. i. Phospho-Thr34, the site phosphorylated by PKA, is dephosphorylated by both PP2A and PP2B (calcineurin) [56]. PP2A is a heterotrimeric enzyme composed of PKA-sensitive PP2A/B56δ and Ca^2+^-sensitive PP2A/PR72 [57]. The heterotrimeric form of PP2A that includes the B56δ subunit is involved in the dephosphorylation of DARPP-32 at Thr34 [58]. Activation of PP2A by PKA leads to the shutting off of D1R/PKA/P-Thr34 DARPP-32 signaling [59]. Thr34 dephosphorylation by PP2B leads to disinhibition of PP1 [24]. ii. Phospho-Thr75 is dephosphorylated by PP1, PP2A and PP2C, and PP2A is the major phosphatase acting on this site [47]. Both PP2A/B56δ and PP2A/PR72 are effective in mediating the dephosphorylation of DARPP-32 at Thr-75 [47]. Dephosphorylation of DARPP-32 at Thr75 relieves the inhibition of PKA [22]. iii. Phospho-Ser97 DARPP-32 is dephosphorylated by PP1 and PP2A. Dephosphorylation of DARPP-32 at Ser97 by PP2A causes nuclear localization of DARPP-32 [60]. However, the effect of PP1 on the dephosphorylation of P-Ser97 DARPP-32 and the subsequent functions are not yet well defined. iv. Phosphor-Ser130 DARPP-32 is dephosphorylated by PP2A and PP2C. The phosphorylation of DARPP-32 at Ser130 stimulated by CK-1 has been implicated in the biological action of mGluR1/5 receptors [61]. However, the biological roles of PP2A and PP2C in dephosphorylation of DARPP-32 at Ser130 have not yet been identified.

### Alteration and activity changes of darpp-32 in animal models of schizophrenia

As described above, it has long been suggested that brain mesocorticolimbic dopaminergic system dysfunction, expressed by increase in dopamine levels or dopamine receptor sub-types expression levels, are involved in the pathogenesis of schizophrenia. Based on this theory, current antipsychotic drugs act as the pharmacological antagonists of D2R. The schizophrenic behavior may be expressed in a specific animal phenotype and may be analyzed under controlled conditions in the laboratory. For example, amphetamine, by increasing synaptic monoamine levels, can induce psychotic-schizophrenic like symptoms including reduced social behavior [62, 63], deficits in pre-pulse inhibition and increased locomotor activity [64]. In amphetamine-induced animal model of schizophrenia, the increased total protein levels of CDK5, p35 and p25 proteins resulted in increased CDK5 kinase activity and subsequently increased the phosphorylation of DARPP-32 at Thr75 site in the brain nucleus accumbens [65]. These results indicate that chronic hyperdopaminergic situation may increase the activity of DARPP-32.

The glutamate hypothesis of schizophrenia based on NMDA receptor hypofunction arose from the observation that administration of noncompetitive NMDAR antagonists, such as phencyclidine (PCP) or ketamine, to healthy individuals, induces hallucinations, delusions, negative symptoms and cognitive impairment that mimic schizophrenia, and exacerbates those symptoms in schizophrenic patients [66]. Administration of PCP in mice with conditional knockout of DARPP-32 in the GABAergic medium spiny neurons enhances DARPP-32 phosphorylation at Thr34 in the striatum [67], and the localization of this effect is preferentially in the MSNs expressing D1R of the direct pathway [68]. Conditional knockout of DARPP-32 in the MSNs abolished the motor stimulatory effects of PCP, while the memory deficits induced by PCP were not associated with the expression of DARPP-32 [68].

Considering findings from schizophrenic animal models, we can conclude that increased phosphorylation of DARPP-32 and its downstream effectors have a causative role in schizophrenia. These changes are associated with the behavioral responses in animals, and can be regarded as a surrogate marker of the positive schizophrenic symptoms [68]. This conclusion is also supported by studies indicating that learning is impaired in calcineurin knockout mice, establishing a link between synaptic anomalies and cognitive impairment observed in schizophrenic mice models [69]. Since DARPP-32 is a major substrate of calcineurin, this is another indirect link to its involvement in schizophrenia.

### Alteration of darpp-32 in schizophrenic patients

In human patients with schizophrenia, DARPP-32 was significantly decreased in the dorsolateral prefrontal cortex circuit (DLPFC) [32], A postmortem study showed a significant decrease of DARPP-32 in the DLPFC of patients with schizophrenia, and the decrease of DARPP-32 was associated with dysfunction of dopaminergic neurons [70, 71]. Moreover, the density of DARPP-32-positive neurons was significantly decreased in layers II-V of the DLPFC [72]. By contrast, the level of phosphorylated DARPP-32 (Thr34) in neurons of layer V of DLPFC was significantly higher [72]. These results are supported by the findings that both the protein level of DARPP-32 and the phosphorylation of DARPP-32 (Thr34) were found significantly lower in the brain superior temporal gyrus of the schizophrenic patients [73]. The density of DARPP-32-positive neurons in the layers II and III of the brain rostral agranular insular cortex was significantly decreased in the schizophrenic group compared with the healthy control group [74]. Cumulatively, these findings indicate that the levels of DARPP-32 protein expression and phosphorylation are decreased in the prefrontal cortex of schizophrenic patients. Interestingly, the other form of DARPP-32, the truncated DARPP-32 protein, is significantly increased in the DLPFC and caudate of patients with schizophrenia [36, 37]. Although no explanation is available, these data support the hypothesis that prevalence of DARPP-32 is associated with the pathogenesis of disease in the schizophrenic patients.

Unfortunately, there are on this topic inconclusive reports as well. No change in DARPP-32 protein levels were found in schizophrenic prefrontal and cingulate cortex and T lymphocytes of elderly patients with schizophrenia [75, 76]. Mutation analysis of DARPP-32 gene indicate that it is not a major susceptibility gene for schizophrenia [77]. In the sight of genetic analyses, no allelic, genotypic or haplotypic association between DARPP-32 and schizophrenia was found in the Chinese population [78], which was consistent with the findings that DARPP-32 are unlikely to be the risk factor of schizophrenia in the Japanese and Malay population [79, 80]. The explanations for these conflicting results remain to be elucidated. It is possible that DARPP-32 contributes to schizophrenia by interacting with other genes, such as dopamine D2 receptor gene and ankyrin-repeat containing kinase 1 gene [36] or other possibilities addressed in the next paragraphs.

### Effects of antipsychotic drugs on darpp-32 signaling

Dopamine D2R is the main target of antipsychotic drugs used for schizophrenia therapy. Chronic blockade of either D1R by SCH-23390 or D2R by raclopride does not increase the level of expression of DARPP-32 protein in specific rat brain regions, including striatum, thalamus, hippocampus and frontal cerebral cortical pole [81]. However, blockade of D2R by typical antipsychotic drugs such as the butyrophenone haloperidol, or the newer antipsychotic drug clozapine, increased the phosphorylation of DDARPP-32 at PKA site [82]. These data indicate that typical or atypical antipsychotic drugs have no effect on the protein or mRNA expression of DARPP-32, but they increase the phosphorylation of DARPP-32. Interestingly, both psychostimulant and antipsychotic drugs have similar effects on the phosphorylation of DARPP-32 at Thr34 site. Chronic administration of psychostimulant cocaine increases the phosphorylation of DARPP-32 (Thr34) and the expression of transcription factor DeltaFosB in the ventral striatum [83]. Paradoxaly, administration of D2R antagonist haloperidol also increases the phosphorylation of DARPP-32. Since these drugs have opposing behavioral and clinical effects, this paradox may be explained by the drug effect on different types of neuronal pathways or different brain structures. Cell-type specific analysis of DARPP-32 phosphorylation in striato-nigral and striato-pallidal neurons indicated that cocaine selectively enhanced the phosphorylation of DARPP-32 at Thr34 selectively in striato-nigral neurons, while haloperidol stimulated the phosphorylation of DARPP-32 at Thr34 only in striato-pallidal neurons [84]. Therefore, cocaine effect is exerted through D1R-mediated activation of AC, which is also called direct MSN pathway. In contrast, the effect of haloperidol is exerted through blockade of D2Rs and disinhibition of AC in MSNs, which is called the indirect pathway [84, 85]. Moreover, in the striato-pallidal neurons, blockade of D2Rs by antipsychotic drugs inhibited adenosine A2 receptors-mediated activation of cAMP signaling, leading to increased level of intracellular cAMP and activation of PKA [85, 86].

Nurr77 is an immediate-early gene of the nuclear receptor super family, which is highly expressed in the brain prefrontal cortex, dorsal striatum, and nucleus accumbens. Several investigations indicate that Nurr77 mediates biological effects of dopamine receptors blockade or stimulation. Nur77 is also involved in the antipsychotic motor lateral effects of antipsychotic agents [87, 88]. It has been shown that haloperidol increases the expression of Nur77 in D2R- but not D1R-expressing neurons, and importantly, Nur77 induction by haloperidol was prevented in T34A DARPP-32 knock mutant mice model, indicating that expression of Nur77 in striato-pallidal neurons is mediated by DARPP-32 -dependent regulation of PP-1 [87].

### Potential relationships between neurotrophins and darpp-32 gene polymorphisms

Neurotrophins, particularly brain-derived neurotrophic factor (BDNF) and nerve growth factor (NGF), are crucial mediators involved in the regulation of neuronal survival, proliferation, differentiation, neurodevelopment and maintenance of neurons in the central and peripheral nervous systems [89–91]. The neurodevelopment hypothesis of schizophrenia is based on the findings of: i. decreased plasma and serum BDNF [92] and NGF [93] levels in schizophrenic patients; ii. BDNF promotes the development of brain mesolimbic dopaminergic system, BDNF synthesized by dopamine neurons influences the expression of dopamine D3 receptors [94]; iii. NGF is maintaining and regulating the phenotype and neuronal circuits of cholinergic neurons, and thereby involved in the process of learning and memory [95]. iv. Low expression of NGF in prefrontal cortex and low levels of BDNF in hippocampus, measured in rodent models of schizophrenia, may indicate that they are associated with cognitive impairment during disease progress and its therapy with antipsychotic drugs [95]. Moreover, BDNF received the most attention in suicide research indicating reduced levels in blood cells of suicidal patients and in brains of patients who committed suicide [96] and a strong link was found between suicidal behavior in schizophrenic patients and BDNF functional polymorphism [97]. Therefore, genes involved in the dopamine pathway such as BDNF and DARPP-32 and their possible association, are biologically plausible candidates in schizophrenic patients with suicide attempts.

The most extensively single nucleotide polymorphism (SNP) studies of BDNF gene is rs6265, which produces a G/A amino acid substitution (valine to methionine) at codon 66 (Val66Met) [98]. This functional polymorphism caused behavioral changes, including anxiety and depression in a genetically engineered mice and may impair human memory and hippocampal function [99]. Preliminary association studies between the functional SNP Val66Met and schizophrenia have generated conflicting results. A positive association was found in a family study in Italian subjects [100]. Patients carrying at least one copy of the minor allele Val66Met functional BDNF marker have attempted suicides compared to patients with other genotypes and non-suicidal patients [101]. In another study it was found that BDNF Val66Met allele is an independent predictor or risk factor of suicide attempts of depressed patients [96]. In a recent meta-analysis study, a significant association was found between BDNF Val66Met polymorphism and schizophrenia, with pooled results indicating no significant evidence for the association of Val/Val and Val/Met genotypes of BDNF gene with disease. However, it was observed that there is a significant association between Met/Met polymorphism and schizophrenia in Asian, European, and Chinese populations, suggesting that the risk of schizophrenia in these populations with Met/Met genotype, is 9, 26, and 9%, respectively [102]. In line with these findings, a recent study demonstrated association of schizophrenia with the rs6330, rs4839435 and rs734194, rs11466155, rs2072446 functional SNPs of genes encoding NGF and NGF receptor [103]. Reduced expression of DARPP-32 has been observed in the postmortem brain of schizophrenic patients [32]. This finding was suggested to be related to the abnormalities observed in patients with schizophrenia, e.g., activation, neostriatal volume, and functional connectivity in the prefrontal cortex [104]. Five molecular variants of DARPP-32 were identified: 1. a C-to-G substitution at nucleotide position −2036 (g.-2036C>G); 2. Absence of G in the untranslated exon 2 (g.1238delG); 3.a G-to-A and 4.an A-to-G substitutions in intron 2 (IVS2+31G>A) and intron 6 (IVS6+32A>G), respectively, and 5. deletion of a three-base pair (AGA) in exon 6 that led to the absence of a glutamate at codon 135 (E135del) [77]. In view of increase in variable analyses obtained from studies in world populations of schizophrenic patients, there is a need to examine the association between the BDNF Val66Met and the above five variants DARPP-32 - polymorphism.

Preclinical experiments demonstrated that BDNF elevates the temporal and perhaps spatial expression of DARPP-32 [105], and BDNF receptor (trkB- tropomyosin receptor kinase B)signaling is thought to be the activator of DARPP-32 striatal expression [106]. BDNF^−/−^ mice showed delayed and decreased expression of DARPP-32, while BDNF treatment *in vivo* increases the level of DARPP-32 [105]. Further studies verified that both the phosphatidylinositol 3-kinase (PI3K)/protein kinase B (Akt) /mammalian target of rapamycin (mTOR) and the CDK5/p35 signaling pathways are involved in the regulation of DARPP-32 protein levels by BDNF in MSNs. [107]. The inherent weaknesses of these studies are likely confounded by clinical heterogeneity, effects of chronic illness and medications and have been limited by schizophrenic patient cohort size and the complex genetics involved in this disease. Therefore, future preclinical and clinical studies may significantly clarify the association between DARPP-32, BDNF and other genes polymorphism to elucidate the mechanisms of these genetic associations with schizophrenia illness in human brain [108].

## PERSPECTIVES AND CONCLUSIONS

Dysfunction of dopamine system triggers a shift of the balance between the phosphorylation and dephosphorylation of DARPP-32, which is highly associated with abnormal psychiatric behavior. An adjustment of this imbalance is supposed to be beneficial for the treatment of CNS disorders, including schizophrenia [109]. Stimulation or blocking neurotransmitters’ receptors is a practical pharmacological approach to promote the phosphorylation state of DARPP-32. On the other hand, the activity of DARPP-32 also depends on the level of intracellular cAMP. cAMP level is not only regulated by AC synthesis, but also by phosphodiesterases (PDEs) degradation of this second messanger. Therefore, inhibition of PDEs, such as PDE4, is a alternative strategy to stimulate the phosphorylation of DARPP-32 (Figure 1). Recently, it has been reported that inhibition of PDE4 by the drug rolipram activated cAMP/PKA signaling and enhanced the phosphorylation of DARPP-32 in the frontal cortex [110]. However, approved PDE4 drug inhibitors such as cilomilast and roflumilast cause intolerable side effects, such as nausea and emesis [111]. Novel PDE4 inhibitors, such as chlorbipram and roflupram, which have little emetic potential and found effective as antidepressants and cognitive enhancers in animal experiments [111–113], are promising drugs for the clinical application in schizophrenia by targeting DARPP-32 phosphorylation. To the best of our knowledge, none of these novel PDE4 inhibitors have been tested in schizophrenic patients by clinical trials.

Currently, most of the medical translational studies focused on the phosphorylation state of DARPP-32, and they represent a promising direction [114]. On the other hand, we may also need to pay attention to the protein expression of DARPP-32 in the brain, especially in the brain regions highly related to schizophrenia. Postmortem studies show a significant reduction of DARPP-32 in the schizophrenic brain [70, 71] as also observed in prenatal LPS-induced animal model of schizophrenia [115]. Therefore, from a pharmacological point of view, increasing the expression of DARPP-32 may be beneficial for schizophrenia therapy. It is interesting that BDNF promotes the expression of DARPP-32 through PI3K/Akt/mTOR sigaling pathway [105, 106], enhances neurodevelopment and neural plasticity [116–118] and corrects other deficits reported in schziphrenic patients [119, 120]. We predict that future drug development protocols for schizophrenia will consider small molecules or biologics that specifically target DARPP-32 and its interacting proteins.

In conclusion, in the last three decades, scientific investigations have proved that DARPP-32 acts as a major integrator signaling phosphoprotein, activated by a diverse array of neurotransmitters, such as dopamine, glutamate, serotonin, adenosine, and gamma-aminobutyric acid in the CNS [114]. Importantly, current studies supported the involvement of DARPP-32 in the pathology of CNS disorders. The role of DARPP-32 proteins in schizophrenia has been recently recognized and aroused wide interests to unveil new functions of DARPP-32. Collectively, the present preclinical and clinical findings strongly suggest that DARPP-32 protein is a promising therapeutic target for the treatment of schizophrenia.
